# Examining human reliance on artificial intelligence in decision making

**DOI:** 10.1038/s41598-026-34983-y

**Published:** 2026-02-05

**Authors:** Joe Pearson, Itiel E. Dror, Emma Jayes, Grace-Rose Whordley, Georgina Mason, Sophie Nightingale

**Affiliations:** 1 Department of Psychology, Lancaster University, Lancaster, LA1 4YF UK; 2 Cognitive Consultants International (CCI-HQ), London, UK; 3 Defence Science and Technology Laboratory, Wiltshire, UK

**Keywords:** Computational social science, AI, Decision-making, Bias, Psychology, Human behaviour

## Abstract

The use of Artificial Intelligence (AI) to effectively support human decision making depends on whether humans are willing to trust in, and thus rely on, AI. Understanding human reliance on AI is critical given controversial reports of AI inaccuracy and bias. Furthermore, the erroneous belief that using technology removes biases may lead to overreliance on AI. To examine humans’ reliance on AI, human participants (*N* = 295, *M*_*age*_ = 33.79) judged the authenticity of 80 faces (40 real, 40 AI-synthesized) presented alongside guidance supposedly from humans or from AI. This guidance was correct only half of the time. Participants indicated their confidence in each judgement and completed measures to examine propensity to trust humans and general attitudes towards AI. Participants who received AI guidance and exhibited more positive attitudes towards AI showed poorer discriminability between real and synthetic faces than those with less positive attitudes towards AI. For participants who received human guidance, level of trust in humans did not affect discriminability. Therefore, AI-derived guidance may be uniquely placed to engender biases in humans, leading to less effective decision making. To ensure successful human-AI decision making partnerships, more research is needed to understand precisely how humans use AI guidance in various contexts.

**Supplementary Information:**

The online version contains supplementary material available at 10.1038/s41598-026-34983-y.

## Introduction

Advances in technology and access to “big data” have allowed for the expansion of Artificial Intelligence (AI) to help with human decision-making. There are, potentially, significant benefits to using AI to support human decision-making, including saving time, improving accuracy, and reducing bias. Indeed, one need only consider the use of AI by various music and film streaming platforms and dating sites to grasp how accepted and *expected* the use of such technology has become^1,2^. However, in recent years, the use of AI in certain contexts has led to much controversy, with reports indicating inaccuracy and unfairness; notably showing biases against certain demographic groups^3^. For example, the COMPAS tool used in many US states to predict a defendant’s risk of recidivism has been shown to be biased against people of colour^4^. Although there is a growing literature addressing the technical limitations of AI and trying to improve accuracy and fairness, there is insufficient research examining human-AI interactions. Specifically, the impact that guidance provided by AI, and a human decision-maker’s general attitudes towards AI, will have on the accuracy and manifestation of biases in the individual’s decisions.

The human tendency to over rely on technology is not a novel phenomenon. Over-trusting or over-relying on automated systems, including AI, is broadly defined as *automation bias* - a cognitive phenomenon whereby human operators tend to favour recommendations derived from a technological source over their own judgements^5^. One of the earliest critiques of automation came in the context of aviation where questions were raised around the safety of shifting from manual to automated flight-desk functions^6^. Even nearly half a century ago, the pace at which automation in aviation was advancing was described as outstripping the ability to comprehend the consequences for the different demands this change placed on human operators^7,8^. Namely, interacting with automated systems draws on human operators in a different way, shifting demands on human cognition—from active control to more passive monitoring, where the human is expected to intervene in instances that the technology cannot handle^9^. Various aspects of human nature, human understanding of automated systems, and automation optimisation can contribute to the manifestation of automation bias. For example, much research has shown that when supervising automated systems, humans tend to demonstrate a loss of situational awareness which can lead to a failure to intervene to correct any errors (e.g.,^10,11^). Although much work has focused on the aviation sector, concerns about automation bias extend to other domains such as healthcare and military, especially with the relatively recent move to embed AI systems within these areas to assist human decision making^12,13^. Automation bias has long presented a challenge in traditional computer-assisted decision-making and now poses critical concerns in the face of human-AI interaction.

Research has begun to reveal the extent to which human cognition restricts human-AI interactions and negatively impact real-world decision-making. People can, for example, succumb to a fallacy known as *Technological Protection*– the notion that the use of technology will remove biases^14^. This misplaced belief in the impartiality of technology may result in over reliance and misplaced trust in guidance derived from AI^15^. AI frequently provides outputs without the uncertainty cues common to human interaction. Response delays, disfluencies, and rephrasing during human interactions allow humans to gauge the credibility of incoming information. Without these cues, humans encountering AI may mistakenly attribute high confidence and, therefore, trustworthiness to AI outputs^16^. AI is also often portrayed as accessing and utilising all human knowledge^17^. Humans tend to accept, and form stronger beliefs based on, incoming information believed to be from a more credible and knowledgeable source^18,19^. Furthermore, the widespread willingness to discuss and describe AI as anthropomorphic^20^ reflects the known tendency of humans to readily assign human characteristics such as intentionality and—consequently—certainty to AI^21^. Thus, the design and portrayal of AI works with human nature, such that each has the potential to facilitate automation bias and, therefore, drive biased use of AI by human operators.

Importantly, greater reliance on and/or trust in automated technologies engendered by the aspects of human nature and AI design described above has long been understood to increase use and misuse of such technologies by human operators^22–25^. Indeed, frequent use and, therefore, familiarity with AI that is designed to support humans during decision-making tasks, may lead to problematic over-reliance on AI such that it is treated as an entirely autonomous decision-maker. One such example is the crash of Continental Flight 3407 in February 2009, wherein increased automation of cockpit procedures led to failures among crew to pay attention^26^. The consequences of human reliance on, and misplaced trust in, biased AI can also be seen in the use of Automated Fingerprint Identification Systems (AIFS). Human operators tend to over-rely on these systems to provide the most likely match at the top of the candidate ranking list, hence they make more false positive decisions on candidates on the top of the list and, conversely, more false negative decisions on candidates further down the list^27,28^.

Currently there is a lack of research and understanding about how humans and AI interact in decision-making tasks – do humans use AI effectively, or do they place too much reliance in the guidance AI provides, thereby reinforcing and legitimising cognitive biases within decision making? Importantly, to isolate the specific effect of AI, the research reported here included both AI and human guidance to allow comparison of results across these input types. An understanding of the effect of AI guidance on decision-making is important to ensure maximal benefit from technological advances while avoiding pitfalls. This research will highlight potential ways to use AI more effectively, for example how to reduce bias and under what circumstances are biases most likely. As such, beginning to understand the characteristics of AI-created bias will allow a more informed appreciation of how AI can benefit strategic and operational planning, while a greater understanding of perceptions and trustworthiness of AI in the decision making process will increase transparency. Overall, understanding the impact of AI on human cognitive bias is crucial before AI is widely deployed and integrated into human decision-making procedures. The aim of the current research is to provide the initial steps in gaining such an appreciation.

To do so, this research examined whether humans use AI and human guidance in a useful way – that is, rely on the guidance if it is accurate and dismiss it if it is inaccurate. We used a relatively simple decision-making task – determining whether a face is real or synthetic^29^. There are two clear benefits to using this task: (1) the stimuli set is already validated and (2) previous research provides baseline accuracies allowing us to select facial images that, although difficult to classify, can be accurately classified by most people (accuracy range of 64–84%). The study employed a mixed experimental design: a between-subjects manipulation wherein participants received either human or AI guidance; within-subjects manipulations of stimulus authenticity (real vs. synthetic faces) and guidance accuracy (correct vs. incorrect).

### Research questions

1. Will participants who receive guidance (from AI or humans) show a similar decision accuracy to a baseline control comparison group (from^29)^?
2. How will response accuracy (correct identification of faces as either real or synthetic) be affected when participants are given incorrect guidance vs. correct guidance?
3. Will participants who receive AI guidance provide more responses consistent with that guidance than participants who receive human guidance?

## Method

### Participants

Participants were recruited via Prolific, an online participant recruitment platform with over 130,000 members vetted to take part in research studies. Prolific users were eligible for participation if they: reported themselves to be fluent in English, have > 95% approval rating, use a desktop computer/laptop with screen size > 1024 × 768 pixels, ≥ 18 years-of-age, and have normal/corrected-to-normal colour vision. A sensitivity power analysis appropriate for two-way ANOVA showed that a sample size of 274 yields a power of 0.80 for a small-to-medium effect size of 0.17 and an *α* of 0.05. Data were collected from 322 Prolific users (26 participants’ data were removed due to: device/operating system check = 9, vision check = 2, attention checks = 9, guidance use check = 2, withdrawn consent = 3, non-complete = 1). Following data cleaning, a final sample of 295 individuals (*M*_age_ = 33.79, *SD* = 10.76) remained. Of these 295 participants, 182 identified as male, 109 as female, 2 as non-binary/genderqueer/agender/gender fluid, 1 as transgender male, while 1 preferred not to say. Additionally, 209 self-reported as White, 58 Black, 12 Mixed, 11 Asian, and 5 Other. Participants were paid £5 upon completion of the experiment, and the amount paid did not depend on a participant’s performance in the face classification task.

### Materials

#### Stimuli

The real and synthetic faces used in this study were taken from Nightingale and Farid (2022;^29^), where a stimulus set of 400 real and 400 synthetic faces was created. Thus, the stimuli used here have been previously validated and have baseline accuracies, allowing us to select stimuli that fall within a certain mean accuracy range (64–84%) that, although difficult to make judgements about, can be accurately categorised as real or synthetic by most people. This accuracy range yielded an available stimulus set of 156 faces (102 real, 54 synthetic). For the current study, 80 stimuli (40 real, 40 synthetic) were selected.

**Table 1:** Number of real and synthetic faces of each available gender and ethnicity.

Gender/ethnicity	Real		Synthetic	
	Count	Mean (SD)	Count	Mean (SD)
Male	20	0.73 (0.06)	19	0.73 (0.06)
Female	20	0.72 (0.05)	21	0.71 (0.06)
Black	11	0.72 (0.06)	16	0.72 (0.06)
East Asian	6	0.72 (0.06)	14	0.71 (0.06)
South Asian	12	0.73 (0.06)	9	0.73 (0.05)
White	11	0.72 (0.06)	1	NA
		**0.71 (0.06)**		**0.71 (0.06)**

Mean and standard deviation (SD) accuracy data derived from^29^.

Whereas stimuli were selected to represent a diverse population, the number of real and synthetic faces available within the prescribed accuracy range made equal representation of gender (male, female) and ethnicity (Black, East Asian, South Asian, White) impossible. The distribution of real and synthetic stimuli across gender and ethnicity identifiers was completed as evenly as possible according to the first author’s discretion (Table 1). The full stimuli selection protocol is available in supplementary materials (SM) 1.

#### Guidance information

**Fig. 1:**
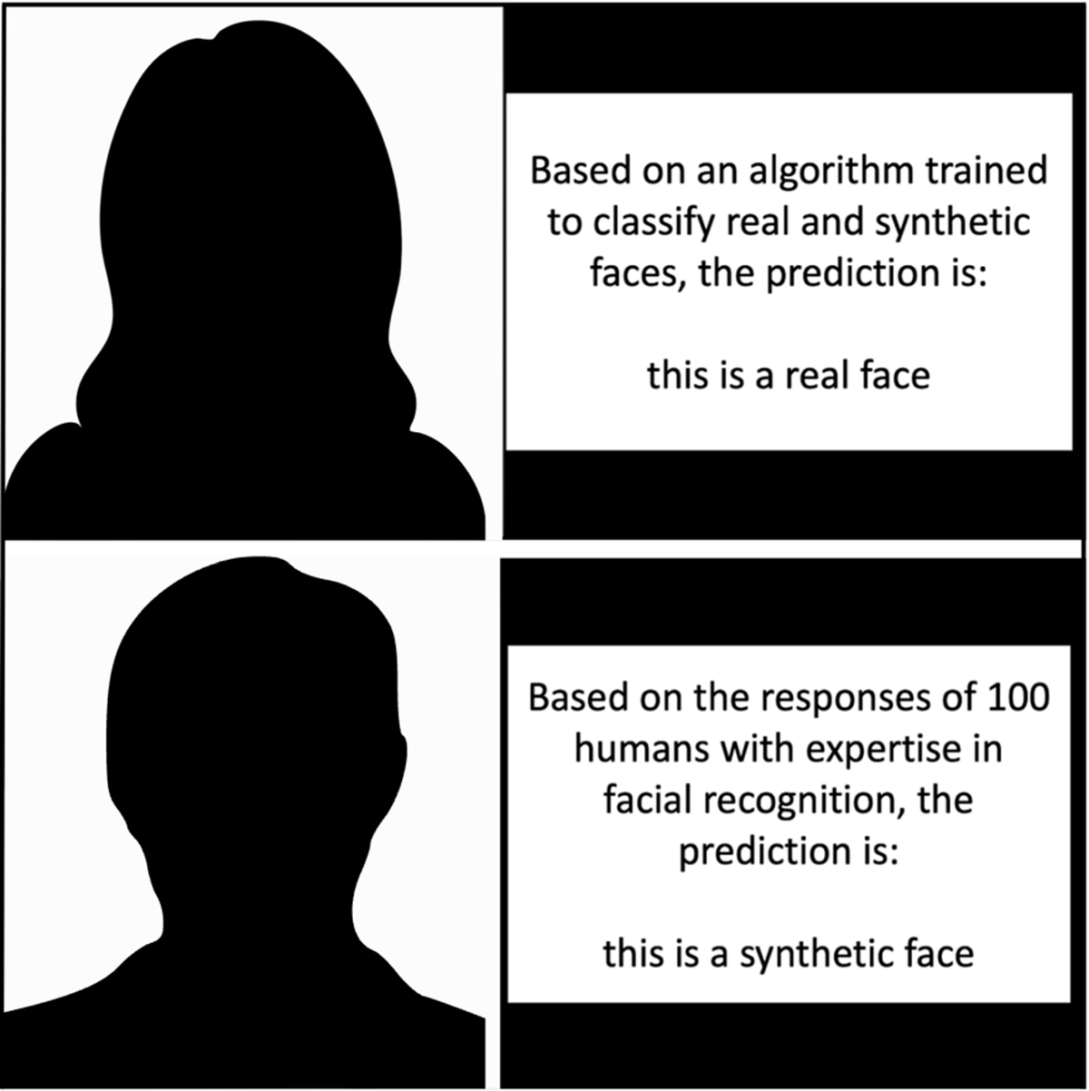
Example stimuli with facial images shown as silhouettes due to licencing permissions: top = synthetic face, AI condition; bottom = real face, human condition. Real faces were obtained from Flikr-Faces-HQ Dataset^30^, made available by NVIDIA Corporation under Creative Commons BY-NC-SA 4.0 license. ‘Prediction’ is favoured over alternatives (e.g., ‘conclusion’) since it suggested an estimate that cued participants to evaluate the stimuli before providing a response. Due to licensing permissions, facial images are shown here as silhouettes to illustrate where the real/synthetic faces appeared.

Each face was paired with correct or incorrect human or AI guidance information, producing standardised information cards (Fig. 1). In both the human and AI guidance conditions, half of the cards provided correct guidance information (e.g., stated a real face was real) and half provided incorrect information (e.g., stated a real face was synthetic). In both the human and AI conditions, ‘A’ and ‘B’ streams were constructed to counterbalance the appearance of stimuli alongside correct or incorrect information (i.e., if a face appeared alongside correct guidance in A, it appeared alongside incorrect guidance in B). All guidance streams included all 80 faces, while 20 of each type of face (real or synthetic) were presented alongside correct guidance and 20 alongside incorrect guidance. Stimuli appeared in a random order, and participants were unaware of the manipulation of real vs. synthetic faces and correct vs. incorrect guidance.

#### Face classification task

Participants responded to all 80 faces. In each trial participants indicated if the face was of a real person or if it had been artificially synthesized. They reported their level of confidence in each judgement using a Likert scale (1 = not at all confident, 5 = extremely confident).

#### Attention check stimuli

To ensure participants engaged with the face classification task, four attention check trials were presented in the first two thirds of the study. These were synthetic faces containing various errors such as missing, misshapen, or discoloured features. To ensure participants knew what these attention check images might look like, they were given a description of the types of errors they could reasonably expect and were also shown four examples of poorly synthesised faces. Participants were informed that these erroneous images constituted attention checks following the same presentation format as the experimental stimuli, and were screened out from the study if they failed to correctly identify at least three of the four attention checks.

#### Human trust scale

Participants completed the human trust scale (SM 2), a 17-item questionnaire drawing on items from several other scales^31–36^ to measure participant beliefs about others’ honesty and trustworthiness. Items are scored from 1 (Strongly Disagree) to 5 (Strongly Agree), and items 7, 8, 9, 10, 11, 12, and 13 are reverse-coded. The score for each item is averaged together to form a continuous measure of generalised trust, such that higher scores indicate greater trust in humans. Example items include: “Most people are basically honest”, “Most people are trustworthy”, and “I usually trust people until they give me a reason not to trust them”. Confirmatory factor analysis (CFA) was used to determine the construct validity of this composite measure of human trust. Internal consistency for the human trust scale used in data analysis was excellent (Cronbach’s α = 0.89, 95% CI [0.87, 0.91];^37^.

#### General attitudes towards artificial intelligence scale (GAAIS)

Participants also completed the GAAIS^38^, a validated 20-item scale assessing attitudes towards AI across positive and negative subscales. Positive subscale items – e.g. “Artificially intelligent systems can help people feel happier” – and negative subscale items – e.g. “I find Artificial Intelligence sinister” – are scored from 1 (Strongly disagree) to 5 (Strongly agree), but negative subscale items (3, 6, 8, 9, 10, 15, 19, 20) are reverse-coded. Separate overall scores for the positive and negative subscales are computed by taking the mean score of each set of items. The higher the score on each subscale, the more positive the attitude toward AI. Both the positive and negative subscales demonstrated excellent (α = 0.91, 95% CI [0.89, 0.92]) and good (α = 0.85, 95% CI [0.83, 0.88]) internal consistency respectively.

#### Guidance use check

Participants completed a final survey item examining how much they used the guidance to inform their judgements on the face classification task. Participants indicated whether they (1) read the guidance information and always used it to help them decide if each face was real or synthetic, (2) sometimes used it, (3) read the guidance information but did not use it, or (4) did not read the guidance information. Individuals who reported not having read the guidance were excluded from data analysis (*n* = 2).

### Procedure

This study was constructed and completed using Qualtrics and published online via Prolific. Before taking part, participants were informed of the study’s purpose, their right to withdraw and payment details, and researcher contact information. Informed consent was obtained electronically, after which participants were subject to a device- and vision-check and advised that use of an ineligible device would result in non-payment. Before the experimental task began, participants saw several example stimuli and completed three practice trials to ensure they understood the task. The practice trials included a real, synthetic, and attention check face, and participants received feedback on each of their responses. Participants were then informed they would see 84 faces alongside guidance that may be of use, although they were not informed that (1) the guidance had been fabricated for the purposes of the study, (2) they had been randomly assigned to one of two experimental conditions, or (3) the presented stimuli had been deliberately balanced.

Participants were randomly assigned to one of the four counterbalanced guidance streams, wherein they completed the experimental task outlined above. Following the experimental task, participants also completed the human trust scale and GAAIS. Finally, participants provided age, gender, ethnicity, and guidance use information. Upon completion participants were fully debriefed. The previously undisclosed experimental manipulations were made clear, as were the true aims of the research. Participants were also reminded of their right to withdraw their data from the study and given the option to do so.

### Analyses

Independent samples t-tests were conducted to determine differences in response accuracy and consistency between human and AI guidance conditions. A one-sample t-test was performed to compare accuracy scores for both human and AI guidance groups with a baseline control group^29^. One- and two-way ANOVAs were carried out to identify differences in response accuracy and consistency at different levels of self-reported guidance use within and across guidance conditions. For these analyses, accuracy scores reflect the proportion of experimental trials a participant classified correctly, while consistency scores reflect the number of trials in which a participant responded in line with the guidance provided (regardless of accuracy).

Signal detection analyses assessed classification accuracy and response bias using indices of discriminability and criterion shift (*d’* and *c* respectively^39,40^). Independent samples t-tests on *d’* and *c* values compared face classification task performance between guidance conditions. Linear regression analyses assessed the influence of human trust scale and GAAIS positive and negative subscale scores on *d’* and *c*.

Ethical approval was granted by Lancaster University’s Faculty of Science and Technology Research Ethics Committee (FST-2023-3241-RECR-3) and Ministry of Defence Research Ethics Committee (2213/MODREC/23), and this experiment was performed in accordance with relevant guidelines and regulations. These methods and planned analyses were preregistered at 10.17605/OSF.IO/SRTHP. Several additional exploratory analyses not preregistered were conducted and have been identified below. Data tidying was completed using R (Version 1.4.1106, RStudio Team, 2021) and Microsoft Excel, and all analyses were completed in R.

## Results

Independent samples t-tests indicated no significant differences in response accuracy between counterbalanced human and AI guidance streams. Consequently, both sets of A and B guidance streams were collapsed into one human and one AI guidance group (see SM 3 for preliminary visualisations (Figure S1) and analyses).

### Response accuracy and consistency

Table 2 shows mean response consistency for each guidance group. Individuals were more inclined to make judgements consistent with the guidance provided when it correctly classified faces as real or synthetic, and we did not find differences in guidance use across demographic characteristics (SM 4 Table S1). A paired-samples t-test was conducted to compare mean consistency scores for correct and incorrect guidance information, revealing a significant difference between the two (*t*(294) = 29.74, 95% CI = [12.17, 13.89], *p* < .001). The effect size (Cohen’s d^41^), of 1.73 indicates a large effect. It seems reasonable to conclude, therefore, that participants used the guidance strategically, relying on it more often when it was useful but disregarding it more frequently when it was not.

**Table 2:** Mean and standard error (SE) consistency scores (counts) for stimuli with correct and incorrect guidance, and all stimuli, across guidance conditions.

Guidance group	Correct (out of 40)	Incorrect (out of 40)	Correct & Incorrect (out of 80)
	Mean (SE)		
AI	30.50 (0.36)	17.90 (0.58)	48.40 (0.76)
Human	30.20 (0.35)	16.70 (0.51)	46.90 (0.59)
	**30.30 (0.25)**	**17.30 (0.38)**	**47.60 (0.48)**

A Mann-Whitney-Wilcox test, appropriate for non-normally distributed data, indicated no significant difference in response consistency between human and AI guidance groups (w = 11509, *p* = .37; human mean = 46.90, AI mean = 48.40). An independent samples t-test revealed no significant difference in response accuracy between human and AI guidance (*t*(293) = − 0.99, 95% CI = − 0.03, 0.01], *p* = .32; human mean = 0.67, AI mean = 0.66). It appears that the accuracy with which individuals correctly classified real and synthetic faces, and the extent to which they classified such faces consistently with the guidance, did not change with the guidance source. Additionally, a one-sample t-test indicated no significant difference (*t*(294) = -1.28, 95% CI == [0.65, 0.67], *p* = .20) between overall response accuracy (0.66) and a baseline accuracy level of 67%^29^.

A one-way between-subjects ANOVA revealed a significant effect of level of guidance use (*Always used* vs. *Sometimes used* vs. *Did not use*) on response accuracy (*F*(2, 292) = 11.71, *p* < .001). The effect size, as measured by generalised eta^2^ ($\:{\eta\:}_{g}^{2}$), was 0.07 (small effect). Pairwise comparisons using the Tukey method (Table 3) revealed the *Always used* group’s mean accuracy was significantly lower than the *Did not use* (coefficient estimate = 0.08, 95% CI = [0.04, 0.12], *p* < .001), and *Sometimes used* groups (0.05, 95% CI = [0.02, 0.08], *p* < .001). A one-way ANOVA examining the effect of guidance use level on response consistency revealed a significant effect of guidance use level (*F*(2, 292) = 8.34, *p* < .001, $\:{\eta\:}_{g}^{2}$ = 0.05). Pairwise comparisons using the Tukey method (Table 3) revealed that the *Always used* group mean consistency was significantly greater than the *Did not use* group (6.15, 95% CI = [2.55, 9.75]), as was the *Sometimes used* group (4.33, 95% CI [1.22, 7.44], *p* = .003).

**Table 3:** Mean and standard error (SE) response accuracy (%) and consistency scores (counts) across guidance use levels.

Guidance use level	Accuracy	Consistency		
	All stimuli		Correct guidance	Incorrect guidance
	Mean (SE)			
Always used	0.62 (0.01)	49.70 (1.26)	29.60 (0.64)	20.10 (0.90)
Sometimes used	0.67 (0.01)	47.90 (0.57)	30.80 (0.30)	17.10 (0.46)
Did not use	0.70 (0.02)	43.50 (0.61)	29.60 (0.54)	14.00 (0.76)

Individuals who reported using the guidance information at their own discretion or not at all performed better than those who claimed to have always used it. This result is not surprising given that adherence to all guidance would yield just 50% accuracy. Of particular interest, though, is the discrepancy between the mean total response consistency of those individuals reporting to have always used the guidance, and the expected consistency of this group. Always using the guidance should yield a consistency score of 80, since judgements made by these individuals are made in line with the available guidance regardless of whether it is correct. It seems, therefore, that some participants misremembered their reliance on the guidance. Visualisations (SM 5, Figure S2) and analyses (SM 5) examining the effect of the interaction between guidance use level and guidance stream and response accuracy and consistency revealed non-significant effects on both response accuracy (*F*(2, 289) = 0.82, *p* = .44) and consistency scores (*F*(2, 289) = 0.30, *p* = .74).

### Signal detection analyses

*d’* and *c* (computed in R using the *psycho* package^42^), represent sensitivity to the difference between real and synthetic faces, and criterion shift (inclination to respond more in one direction than another). These values are derived from counts of hits (correct classification of a face when guidance is correct), correct rejections (correct classification when the guidance is incorrect), misses (incorrect classification when the guidance is correct), and false alarms (incorrect classification when the guidance is incorrect,^43^).

Table 4 shows mean *d’* and *c* scores for human and AI guidance groups and for the entire dataset. A *d’* value of zero indicates no ability to distinguish between real and synthetic faces, and a value of 3 represents close to perfect discrimination. For *c*, a value of zero indicates no response bias (equally likely to respond ‘real’ or ‘synthetic’), negative values indicate that an individual responds ‘real’ more often, and positive values indicate that an individual responds ‘synthetic’ more often. Mean *d’* (.90, 95% CI [.84, .97]) and *c* (-.28, 95% CI [-.33, − .24]) values suggest participants showed an ability to distinguish between real and synthetic faces but a tendency toward responses of ‘real’. To determine if *d’* and *c* values are significantly different to 0, one-sample Wilcox t-tests were carried out. These tests revealed that at the dataset level (*v* = 41986, 95% CI = [-.15, .10], *p* < .001, Cohen’s *d* = 1.62) and in each guidance group *d’* was significantly above 0 (Human: *v* = 11430, 95% CI = [.83, 1.01], *p* < .001, *d* = 1.52; AI: *v* = 9682.50, 95% CI = [.81, .96], *p* < .001, *d* = 1.80). The same was observed for a series of one-sample Wilcox t-tests performed on *c* data at the dataset (*v* = 1813.50, 95% CI = [-.27, − .21], *p* < .001, Cohen’s *d* = − .81), Human (*v* = 593, 95% CI = [-.26, − .19], *p* < .001, *d* = − .83) and AI (*v* = 334, 95% CI = [-.31, − .21], *p* < .001, *d* = − .81) levels. Participants displayed a significantly better than chance ability to distinguish between real and synthetic faces, but a significant bias toward identifying faces as ‘real’. These findings are supported by the positive skew in *d’* distribution and substantial negative *c* distribution illustrated in Figure S3 (SM 6).

**Table 4:** Mean and 95% confidence intervals (CI) of *d’* and c values for each guidance stream.

Guidance stream	d’	c
	Mean (95% CI)	
Human	0.94 (0.84, 1.03)	− 0.27 (-0.31, − 0.21)
AI	0.87 (0.79, 0.95)	− 0.32 (-0.38, − 0.25)
	**0.90 (0.84**,** 0.97)**	**− 0.28 (-0.33**,** − 0.24)**

Mann-Whitney-Wilcox tests appropriate for non-normally distributed data revealed no significant difference in *d’* (*w* = 10564, 95% CI = [-0.15, 0.10], *p* = .69) or *c* (*w* = 10256, 95% CI = [-0.08, 0.03], *p* = .41) scores between guidance groups. It appears, therefore, that the type of guidance an individual receives when making judgements about the nature of real or synthetic faces influences neither their ability to distinguish between the two nor their bias in responding.

To determine if the composite questionnaire used here to examine trust in other humans assesses a latent construct of trust, a confirmatory factor analysis (CFA) using maximum likelihood estimation was conducted (SM 6, Table S2). The model specified one latent variable (trust) underlying all observed indicators (excluding item 11, an attention check). Model fit was determined by examining: Chi-square (X^2^), a measure of overall model fit; Root Mean Square Error of Approximation (RMSEA) and Standardised Root Mean Square Residual (SRMR), measures of how far a model is from perfect fit; Tucker-Lewis Index (TLI) and Comparative Fit Index (CFI), which compare model fit to the worst possible model. The model demonstrated poor fit to the data, as indicated by a significant X^2^ test (X^2^(119) = 967.20, *p* < .001). TLI and CFI scores of 0.69 and 0.73 fall below the commonly accepted threshold of 0.90 for adequate fit^44^, while RMSEA and SRMR values of 0.16 and 0.09 exceed the typical cutoff scores of 0.08. Together, these results indicate that a one-factor structure does not adequately represent the data.

To address the poor fit of this unidimensional model of trust in humans, only those items assessing ‘propensity to trust’ were taken forward to analysis. These four items – ‘I usually trust people until they give me a reason not to trust them’, ‘Trusting another person is not difficult for me’, ‘My typical approach is to trust new acquaintances until they prove I should not trust them’, and ‘My tendency to trust others is high’ – were taken from^31^ and constitute a validated measure of propensity to trust other humans. A confirmatory factor analysis (CFA) using maximum likelihood estimation revealed an acceptable fit for this model. All item loadings were significant (higher than 0.82) and fit statistics were good CFI = 0.99, TLI = 0.96, and SRMR = 0.02, aside from chi square (χ2 (2) = 10.93, *p* = .004) and RMSEA = 0.12 [90% CI 0.06–0.20]. Internal consistency for this new human trust scale was excellent (Cronbach’s α = 0.89, 95% CI [0.87, 0.91];^44^. The average inter-item correlation was 0.67, indicating strong internal consistency among the items. Following recommendations to report and consider the model indices in combination^45–47^, these results suggest that these four items constitute a reasonable measure of propensity to trust other humans. A single measure of human trust was created in accordance with^31^’s recommendations by taking the mean score across the four propensity to trust items. Internal consistency was assessed for both the positive and negative GAAIS subscales. The positive and negative subscales demonstrated excellent (α = 0.91, 95% CI [0.89, 0.92]) and good (α = 0.85, 95% CI [0.83, 0.88]) internal consistency respectively. The items comprising each subscale measure a common construct. The full CFA process is described in SM 6.

To determine if general attitudes towards AI or propensity to trust humans influences task performance, linear regressions were conducted with *d’* and *c* as dependent variables and GAAIS subscales and propensity to trust other humans scale scores as independent variables. A significant effect of the GAAIS negative subscale score on *d’* was identified (*b* = − 0.15, SE = 0.05, *p* = .004). Thus, more positive attitudes toward AI yielded a reduced ability to discriminate between real and synthetic faces. Interestingly, the effect of greater positive attitudes towards AI on discriminability was preserved when the same regression model was fit using AI guidance group data only (*b* = − 0.19, SE = 0.07 *p* = .008), but not when fit using the human guidance group data only. A significant effect of human trust scale on *c* values was observed (*b* = − 0.05, SE = 0.02, *p* = .03), such that a greater propensity to trust other humans predicted a shift toward face classifications of ‘real’. Exploratory analyses were carried out to control for the effects of level of guidance use, guidance stream, participant age, gender, and ethnicity. These parameters were entered into each regression model sequentially, and the impact of increased model complexity on model fit was assessed using ANOVA tests. The full model-building process is described in SM 6.

A linear regression with *d’* as dependent variable and GAAIS positive and negative subscale scores, human trust scale score, and self-reported level of guidance use (*Always used*, *Sometimes used*, *Did not use*) as independent variables was performed. The previously identified effect of GAAIS negative subscale on task performance was preserved (*b* = − .13, SE = .05, *p* = .008). No effect of GAAIS positive subscale or human trust scale scores were observed. A significant effect of self-reported guidance use was observed, so that participants who did not use the guidance showed significantly larger *d’* scores than those who always used it (*b* = 0.38 SE = 0.10, *p* < .001), as did those who only sometimes used it (*b =* 0.25, SE = 0.08, *p* = .001). Lower self-reported guidance use predicted an improved ability to discriminate between real and synthetic faces. The same regression model was fit with *c* score as the dependent variable. The previously identified effect of human trust scale on *c* values was preserved (*b* = − 0.05, SE = 0.02, *p* = .009). Additionally, a significant effect of self-reported guidance use level on *c* value was observed – participants who did not use the guidance showed significantly larger *c* values than those who always used it (*b* = 0.26, SE = 0.07, *p* < .001). Thus, less reliance on guidance predicted a reduced likelihood of classifying faces as real. ANOVA revealed a significant improvement in model fit (*p* < .001) with the inclusion of guidance use level for both sets of regression models.

A linear regression with *d’* as dependent variable and GAAIS positive and negative subscale scores, human trust scale scores, self-reported level of guidance use, and guidance stream as independent variables was performed. No significant effect of guidance stream on *d’* was identified, while the pattern of results for GAAIS subscales, human trust scale, and self-reported guidance use level remained identical to that observed in the previous model. Furthermore, ANOVA revealed a non-significant (*p* = .31) improvement in model fit with the inclusion of guidance stream. The same regression model was fit with *c* score as the dependent variable. No significant effect of guidance stream on *c* score was observed. The pattern of results for the remaining independent variables were identical to those identified in the previous modelling iteration. ANOVA revealed a non-significant (*p* = .16) improvement in model fit with the inclusion of guidance stream. Two final regression models accounting for the influence of participant sociodemographic characteristics on *d’* and *c* scores were created, revealing significant effects of participant gender on *d’* scores (*b* = .17, SE = .07, *p* = .01), and of age on *d’* (*b* = − 0.01, SE = 0.003, *p* < .001) and *c* (*b* = − 0.004, SE = 0.002, *p* = .04). It appears that women showed an increased ability to discriminate between real and synthetic faces, while older participants showed a decreased ability to discriminate between real and synthetic faces and a greater likelihood of classifying faces as real.

**Fig. 2:**
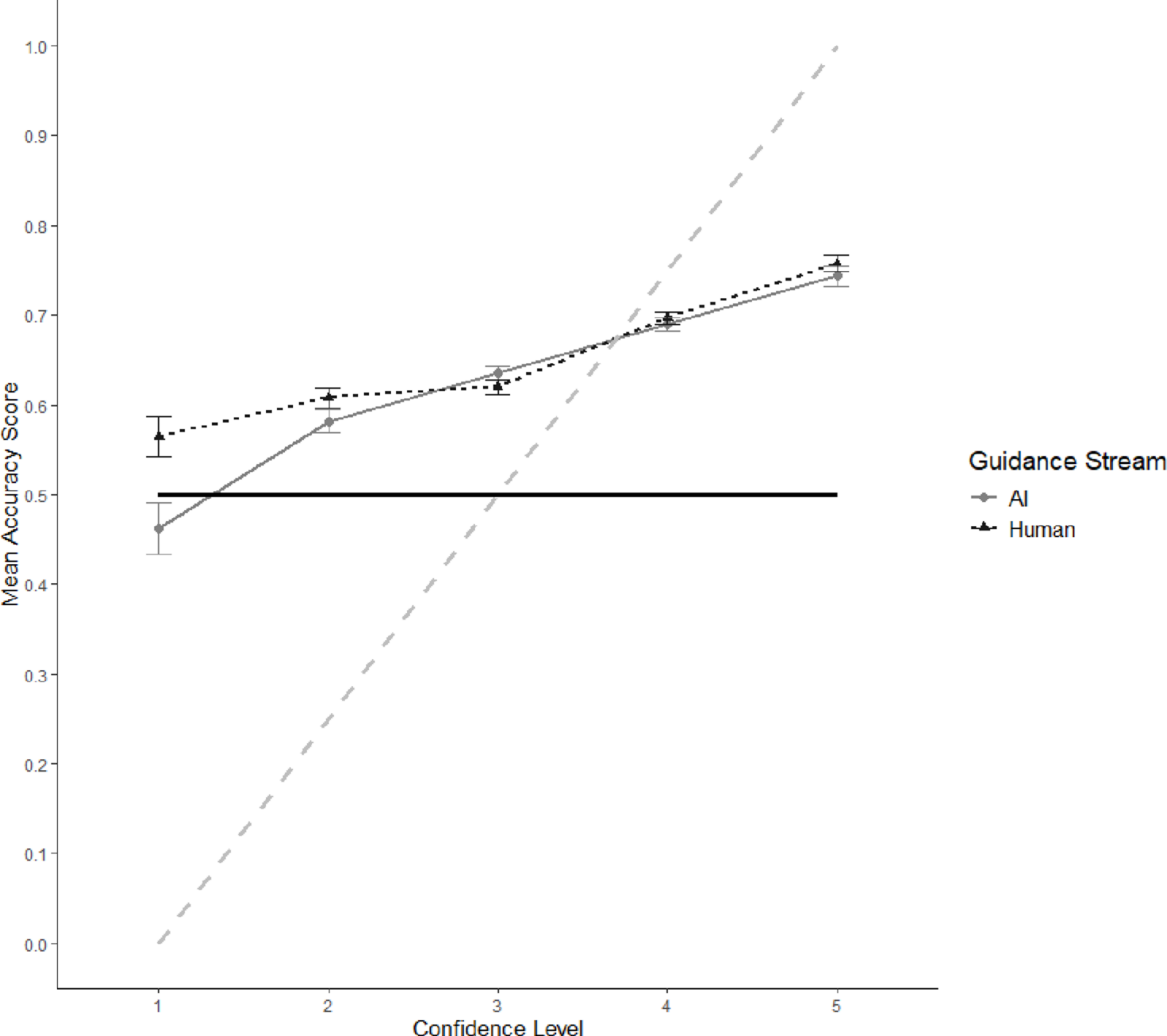
Confidence-accuracy curve of mean accuracy scores (and standard error bars) per level of confidence for AI and human guidance. Diagonal dashed grey line = perfect calibration. Full black line = chance (50% response accuracy).

### Confidence-accuracy calibration

Confidence data collected at each trial (5-point Likert scale: 1 = not at all confident, 5 = extremely confident) were combined with response accuracy data to examine participants’ insights into their decisions. The combined data show the relationship between response accuracy and confidence in judgements (Fig. 2). Those participants who were more confident in their judgements performed better than those who were less confident, regardless of whether they received human or AI guidance.

Additionally, meta-*d’* values were calculated for each guidance condition. Meta- *d’* is a statistical counterpart to the confidence-accuracy relationship visualised above, measuring ‘metacognitive sensitivity’^48,49^. Mean meta-*d’* values of 0.09 (95% CI [0.04, 0.18]) and 0.13 [0.03, 0.18] were observed for the human and AI guidance groups respectively. Positive values occur when high levels of confidence are reported for correct judgements, and low levels for incorrect judgements. In line with the confidence-accuracy curves (Fig. 2) it appears that participants in both guidance groups showed some ability to recognise whether they were making correct and incorrect judgements.

## Discussion

This study examined how reliance upon AI guidance during decision-making influences human judgement and cognitive bias. Participants who received guidance from a supposed human source were as accurate and consistent in their classifications of faces as real or synthetic as those who received supposed AI guidance. Furthermore, a significant response bias towards face classifications of ‘real’ was observed for individuals who received both human and AI guidance, while regression analyses identified reduced task performance (measured by *d’*) for participants with more positive attitudes towards AI than those with less positive attitudes towards AI.

Task performance was not affected by the source of the guidance an individual received. Additionally, it was found that classification consistency *did not* differ significantly with guidance source. This latter finding is surprising given the known tendency for individuals to succumb to the fallacy of Technological Protection^14^. Indeed, we might have expected participants who received AI guidance to show increased consistency with this guidance regardless of whether it was correct or incorrect. Yet, participants showed a similar level of adherence to AI as to human guidance. Furthermore, participants were more inclined to follow guidance when it was correct regardless of its source. Humans appear able to follow guidance when it is correct and disregard it when it is not.

The apparent strategic use of AI guidance is encouraging given what we know about the tendency for over-reliance by humans on potentially biased AI^15^ born out of misplaced positivity towards, and/or trust in, such systems. Thus, during human-AI decision-making interactions it seems that rather than AI protecting against biases – as the fallacy of technological protection might suggest^14^ – it is human decision-makers that work to mitigate biases. In this regard, discrepancies between AI predictions, the subject of a decision (e.g., a real or synthetic face), the specific knowledge of human decision-makers^50^, and prior experience and familiarity with AI systems and their outputs^21,51^ may prevent human operators from following ineffective AI support. Other research, however, highlights the human as the problematic component during human-AI decision-making interactions^52^; the researchers observed in a face-matching task that humans do not perform as well when supported by a simulated automated facial recognition system (sAFRS) as the same sAFRS by itself, due to overturning of correct sAFRS predictions but failures to overturn sAFRS mistakes.

If it is true that humans are a mitigating force against AI biases, one must ask whether it is worthwhile utilising AI in human decision-making at all. At the very least, our focus must be on developing human-AI decision-making interfaces that optimise the regulatory role of humans. Supportive AI of this nature has been of interest to the algorithmic fairness research community for some time^53^, yet how they influence decision-making performance remains unclear^54,55^. If humans are detrimental to human-AI decision-making interactions then the result is the same. Until the issue of AI bias can be resolved – which requires reforms to big data collection practices – it is the human component that we must depend on and enhance to ensure effective AI use.

The stability of response consistency across guidance conditions may also be informed by whether participants relied on the guidance. This seems likely given many participants reported using the guidance only some of the time, whether it was derived from humans or AI. Furthermore, no significant difference in response consistency was observed between those who reported having always used the guidance and those who used it only some of the time. It seems that participants used the guidance as and when they deemed it necessary. This is a sensible and effective strategy highlighting future opportunities for human-AI decision-making partnerships. Occasions under which reliance on available guidance was necessary may have arisen when specific experimental trials presented a difficult choice, such as faces that appear quite but not entirely either real or synthetic. This seems reasonable given previous research identifying increased reliance on advice^56^, and algorithmic advice^57^, by humans when tasks are difficult. Furthermore, automated support system research has highlighted reduced trust in systems deployed on simple tasks^58^. The perceived difficulty of each trial may have determined participant guidance use and may be the mechanism underlying strategic guidance use. Participants likely will have relied on the guidance when they struggled with a decision and disregarded it when confident in their judgements.

Linear regression analyses revealed a significant effect of GAAIS negative subscale score on discriminability amongst individuals who received AI guidance. For individuals with more positive attitudes towards AI, decision-making effectiveness is reduced when they encounter AI guidance. Previous research identifying poorer decision-making amongst humans more frequently using and therefore trusting AIFS^27,28^ seems to support this observation, reaffirming the conclusion that the effectiveness of AI depends on the humans being supported, task difficulty, and guidance quality. This finding bolsters those of previous research identifying the importance of individual differences in trust in AI on human-AI decision-making partnership success, wherein large performance gains have been observed amongst humans re-completing a face-matching task with sAFRS support, especially when they held favourable beliefs about the system^59^. Under these circumstances, the importance of the human at the heart of such interactions is recorded in whether individual differences in trust in technology impedes their acceptance of AI support. That greater trust in humans did not influence discriminability similarly for those who received human guidance suggests AI may be uniquely placed to manifest changes in decision-making ability.

Interestingly, regression analyses showed a significant effect of propensity to trust other humans scale score but not GAAIS score on response bias. Among individuals reporting a greater propensity to trust other humans there is an increased likelihood to identify faces as real. Given that other analyses identified a tendency to classify faces as real regardless of the guidance source, it may be that in a face classification task of this kind participants’ default position is that stimuli depict real faces. For individuals with greater trust in humans, classifications may default in this direction more readily. This is at odds with the previously discussed notions that humans use guidance strategically and that they can act as a regulatory force in human-AI interactions. Why this default position is not overcome and a bias toward classifications of faces as synthetic observed amongst individuals displaying greater positivity towards AI remains unclear. It is worthwhile noting that the CFA fit indices reported here for the propensity to trust other humans scale were mixed, some suggesting a good model fit and others suggesting a weak model fit. The use of this scale is theoretically-driven with it having been developed and validated by existing research^31^, nonetheless, we have cautiously interpreted the human trust results to ensure that the conclusions of this work are valid and useful to the field.

Confidence-accuracy curves suggest that participants in both guidance conditions were able to reflect on their judgements effectively. Positive mean meta-*d’* scores for both guidance groups support this conclusion. That participants demonstrated a good understanding of their capabilities extends similar observations from previous research utilising face stimuli^60^ but contradicts other research identifying unjustified confidence during decision-making^61,62^. The difficulty of this experimental task was controlled to ensure it was possible (by selecting stimuli between a previously identified classification accuracy bracket of 64–84%,^29^). It may be that previous research identifying poor participant insight employed more difficult tasks. This would explain the overall good performance and fair insight displayed by participants.

The various and differing findings observed here mean that more work is required to understand the circumstances under which AI biases manifest, and the role of the human in human-AI interactions. Indeed, given the importance of the human operator being supported in determining AI effectiveness, further investigation of the individual differences influencing the impact of AI on decision making should be prioritised. Future research should manipulate the previously discussed occasions of necessity under which guidance is utilised by humans during decision making, by using decision-making tasks of varying difficulty. This may be achieved by presenting both correct and incorrect guidance with varied accuracy information during a face classification task, yielding scenarios wherein ostensibly highly accurate predictions contradict the accompanying stimuli. Additionally, this experimental paradigm should be applied to various decision-making contexts. Human-AI interactions in, for example, critical military reconnaissance scenarios may manifest biases differently to those in low-demand online experiments.

By developing an increasingly nuanced conceptualisation of human-AI decision-making interactions, and the variation in these interactions across contexts, more effective AI and protocols for their use can be developed. It is imperative, though, that these tools are developed with the best interests of human operators in mind and deployed with fully informed human decision-makers at the heart. Ultimately, AI without human intervention can be useful, but our findings suggest that it is humans who decide how and when.

## Supplementary Information

Below is the link to the electronic supplementary material.

Supplementary Material 1

## Data Availability

The data collected during this research, and the full, anonymised, reproducible R data tidying and analysis code is available at https://osf.io/2p3bf/?view_only=868c92c940c947b894d24ac4b4155607.
