# Supplementary material for: Examining human reliance on artificial intelligence in decision making
Source: Sci Rep. 2026 Feb 5;16:5345. doi: 10.1038/s41598-026-34983-y (PMC12881489; doi:10.1038/s41598-026-34983-y)
Supplement: Supplementary file 1 — Supplementary Material 1 [file 41598_2026_34983_MOESM1_ESM.docx]

**Supplementary Materials**

1. **Stimuli selection protocol.**

- Split accuracy range into four brackets (64-69%, 70-74%, 75-79%, 80-84%)
- Ideally select 1 male (m) and 1 female (f) face for each ethnicity identifier in each accuracy bracket, plus an additional male/female pair from one of the ethnicity identifiers (because there are four ethnicity identifiers – B (Black), W (White), SA (South Asian), EA (East Asian)) to yield 10 stimuli taken from each accuracy bracket, evenly spread across male/female and B/W/SA/EA

Real faces:

- Only 1 EA.F
- Only 4 EA.M
- 64-69%:
  - 1 B.M 1 B.F
  - 1 W.M 1 W.F
  - 1 SA.M 1 SA.F
  - 1 EA.M **0** **EA.F**
- 70-74%:
  - 1 B.M 1 B.F
  - 1 W.M 1 W.F
  - 1 SA.M 1 SA.F
  - 1 EA.M 1 EA.F
- 75-79%:
  - 1 B.M 1 B.F
  - 1 W.M 1 W.F
  - 1 SA.M 1 SA.F
  - 1 EA.M **0** **EA.F**
- 80-84%:
  - **0 B.M**  1 B.F
  - 1 W.M **0 W.F**
  - 1 SA.M **0** **SA.F**
  - 1 EA.M **0 EA.F**
  - The 3 EA.F, 1 W.F, 1 SA.F, and 1 B.M not identified were redistributed throughout the accuracy range as evenly as possible.
  - Additionally, since this dataset only contained 1 EA.F stim, the 3 EA.F still required were split between the other ethnicity identifiers (1 B.F, 1 W.F, 1 SA.F)
- 64-69%:
  - 2 B.M 2 B.F
  - 1 W.M 1 W.F
  - 1 SA.M 1 SA.F
  - 1 EA.M **0** **EA.F**
- 70-74%:
  - 1 B.M 1 B.F
  - 1 W.M 3 W.F
  - 1 SA.M 1 SA.F
  - 1 EA.M 1 EA.F
- 75-79%:
  - 1 B.M 1 B.F
  - 1 W.M 1 W.F
  - 1 SA.M 2 SA.F
  - 1 EA.M **0** **EA.F**
- 80-84%:
  - **0 B.M**  1 B.F
  - 1 W.M **0 W.F**
  - 1 SA.M **0** **SA.F**
  - 1 EA.M **0** **EA.F**
- Additional M/F pair in each bracket to be allocated based on the number of stimuli from each gender & ethnicity already present in that bracket. Additional pair for 80-84% bracket redistributed elsewhere.
- 64-69%:
  - 2 B.M 2 B.F
  - 2 W.M 2 W.F
  - 2 SA.M 2 SA.F
  - 2 EA.M **0** **EA.F**
- 70-74%:
  - 1 B.M 1 B.F
  - 1 W.M 3 W.F
  - 2 SA.M 3 SA.F
  - 1 EA.M 1 EA.F
- 75-79%:
  - 2 B.M 2 B.F
  - 1 W.M 1 W.F
  - 1 SA.M 2 SA.F
  - 1 EA.M **0** **EA.F**
- 80-84%:
  - **0 B.M**  1 B.F
  - 1 W.M **0 W.F**
  - 1 SA.M **0** **SA.F**
  - 1 EA.M **0** **EA.F**

Synthetic faces:

- Only 1 W.F
- No W.M
- 64-69%:
  - 1 B.M 1 B.F
  - **0 W.M** 1 W.F
  - **0 SA.M** 1 SA.F
  - 1 EA.M 1 EA.F
- 70-74%:
  - 1 B.M 1 B.F
  - **0 W.M** **0 W.F**
  - 1 SA.M 1 SA.F
  - **0 EA.M** 1 EA.F
- 75-79%:
  - 1 B.M **0 B.F**
  - **0 W.M** **0 W.F**
  - 1 SA.M 1 SA.F
  - **0 EA.M** 1 EA.F
- 80-84%:
  - 1 B.M 1 B.F
  - **0 W.M** **0 W.F**
  - **0 SA.M** 1 SA.F
  - 1 EA.M **0** **EA.F**
  - The 1 B.F, 2 SA.M, 2 EA.M and 1 EA.F not identified were redistributed throughout the accuracy range as evenly as possible.
- 64-69%:
  - 1 B.M 2 B.F
  - **0 W.M** 1 W.F
  - **0 SA.M** 1 SA.F
  - 3 EA.M 2 EA.F
- 70-74%:
  - 1 B.M 1 B.F
  - **0 W.M** **0 W.F**
  - S SA.M 1 SA.F
  - **0 EA.M** 1 EA.F
- 75-79%:
  - 1 B.M **0 B.F**
  - **0 W.M** **0 W.F**
  - 1 SA.M 1 SA.F
  - **0 EA.M** 1 EA.F
- 80-84%:
  - 1 B.M 1 B.F
  - **0 W.M** **0 W.F**
  - **0 SA.M** 1 SA.F
  - 1 EA.M **0** **EA.F**
- There was only one more SA.M, so the remaining unidentified SA.M reallocated to B.M (since there are fewer B.M than EA.M thus far)
- The 4 W.M required distributed between the other ethnicity identifiers (apart from SA.M because there are no more), 2 B.M and 2 EA.M
- The 3 W.F required distributed between the other ethnicity identifiers, 1 B.F, 1 SA.F, 1 EA.F
- 64-69%:
  - 2 B.M 3 B.F
  - **0 W.M** 1 W.F
  - **0 SA.M** 2 SA.F
  - 5 EA.M 2 EA.F
- 70-74%:
  - 1 B.M 1 B.F
  - **0 W.M** **0 W.F**
  - 2 SA.M 1 SA.F
  - **0 EA.M** 1 EA.F
- 75-79%:
  - 3 B.M **0 B.F**
  - **0 W.M** **0 W.F**
  - 1 SA.M 2 SA.F
  - **0 EA.M** 2 EA.F
- 80-84%:
  - 1 B.M 1 B.F
  - **0 W.M** **0 W.F**
  - **0 SA.M** 1 SA.F
  - 1 EA.M **0** **EA.F**
- Additional M/F pair in each bracket to be allocated based on the number of stimuli from each gender & ethnicity already present in that bracket.
  - No W.F stim remaining, so 2 B.F, 1 SA.F, and 1 EA.F spread across the accuracy range
  - No W.M or SA.M stim remaining, so 2 B.M and 2 EA.M spread across the accuracy range (however only 2 EA.M remaining, and 1 is the exact same accuracy as another EA.M stim, so reallocated to 1 EA.M and 1 EA.F)
- 64-69%:
  - 3 B.M 4 B.F
  - **0 W.M** 1 W.F
  - **0 SA.M** 2 SA.F
  - 5 EA.M 4EA.F
- 70-74%:
  - 1 B.M 2 B.F
  - **0 W.M** **0 W.F**
  - 2 SA.M 1 SA.F
  - **0 EA.M** 1 EA.F
- 75-79%:
  - 3 B.M **0 B.F**
  - **0 W.M** **0 W.F**
  - 1 SA.M 2 SA.F
  - **0 EA.M** 2 EA.F
- 80-84%:
  - 2 B.M 1 B.F
  - **0 W.M** **0 W.F**
  - **0 SA.M** 1 SA.F
  - 2 EA.M **0 EA.F**

1. **Trust scales.**

**The General Attitudes towards Artificial Intelligence Scale (GAAIS).**

The items included in the GAAIS scale along with instructions for use is available in the original publication: Schepman & Rodway (2020): Attitudes towards Artificial Intelligence https://doi.org/10.1016/j.chbr.2020.100014

**Human Trust Scale.**

The human trust scale in our study is a 17-item questionnaire which includes five items from Yamagishi and Yamagishi (1994), four items from Yamagishi (1986), and four items from other scales (Rotter, 1967; Yamagishi, 1988; Glaeser et al., 2000) which measure participants’ beliefs about honesty and trustworthiness of others, in general. We also include Frazier et al.’s propensity to trust scale which includes four items and measures a general willingness to trust others, regardless of social and relationship-specific information.

**Scale:**

Using the following scale, please indicate how much you agree or disagree with the following statements:

1 Strongly Disagree

2 Disagree

3 Neutral

4 Agree

5 Strongly Agree

**Items:**

1.) Most people are basically honest.

2.) Most people are trustworthy.

3.) Most people are basically good and kind.

4.) Most people are trustful of others.

5.) I tend to trust others even if I have little knowledge of them.

6.) Most people will respond in kind when they are trusted by others.

7.) Most people tell a lie when they can benefit by doing so.

8.) Those devoted to unselfish causes are often exploited by others.

9.) Some people do not cooperate because they pursue only their own short-term self-interest.

Thus, things that can be done well if people cooperate often fail because of

these people

10.) If given a chance, most people would try to take advantage of you

11.) Most people are too busy looking out for themselves to be helpful

12.) You can't trust people anymore

13.) When dealing with people, one is better off using caution before trusting them

14.) I usually trust people until they give me a reason not to trust them

15.) Trusting another person is not difficult for me

16.) My typical approach is to trust new acquaintances until they prove I should not trust them

17.) My tendency to trust others is high

A.) Attention Check: I would be grateful if you could select disagree.

**Scoring:**

The score for each item is averaged together to form a continuous measure of

generalized trust. Items 7, 8, 9, 10, 11, 12, and 13 are reverse coded.

1. **Preliminary data visualisation and counterbalanced stream merger.**

Initial plots visualising the distribution of response accuracy data were created. Figure S1 shows histograms of the distribution of response accuracy in each of the four guidance information streams. These plots show reasonably normally distributed response accuracy data in each guidance stream, albeit slightly positively skewed data where the AI_A and HumanB groups are concerned. Figure S1 also shows those respondents identified as outliers. The presence of a ‘hyper-recogniser’ – in this context an individual with the ability to distinguish between real and artificially synthesized faces with extremely high accuracy – in the HumanB guidance stream (95% response accuracy) may account for the aforementioned positive skew.

**Figure S1**

*Distribution of response accuracy data in each counterbalanced guidance stream.*

*
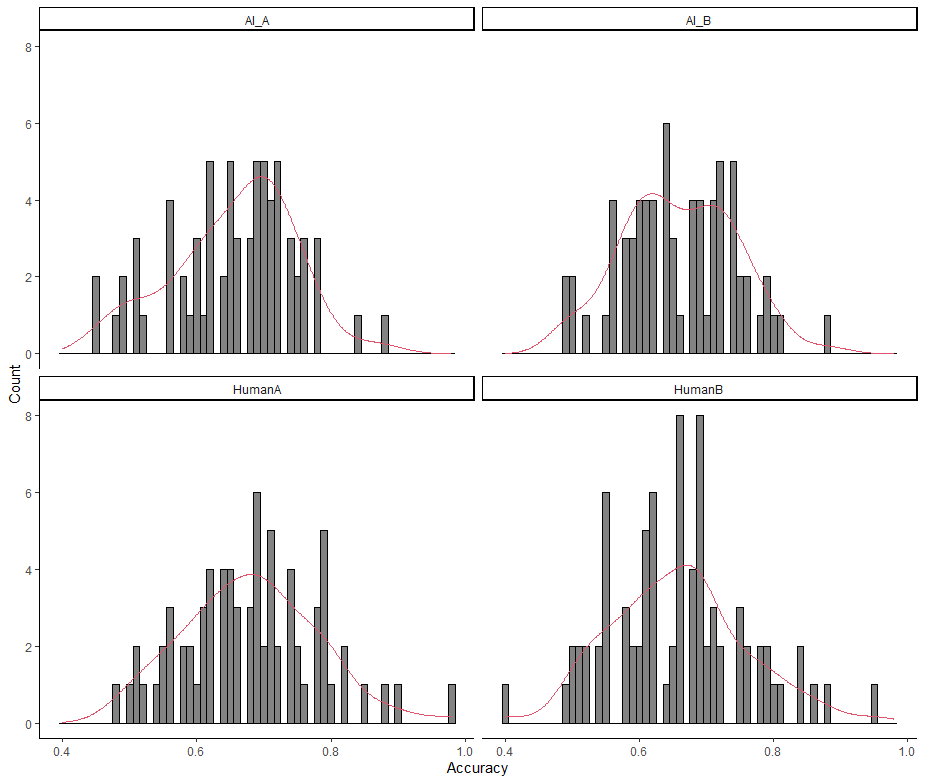
*

The HumanB guidance stream also contains a ‘hypo-recogniser’, with a response accuracy of 40%. Given that complete adherence to the presented guidance information yields a response accuracy of 50%, since half the stimuli were accompanied by incorrect guidance information, a response accuracy of 40% suggests a particular inability to distinguish between real and artificial faces. The HumanA guidance stream also contains a hyper-recogniser (98% response accuracy), although no clear effect on response accuracy distribution is seen. These outliers were included in all planned statistical analyses, since an understanding of the factors driving hyper- and hypo-recognisers’ performance may improve our understanding of how artificially synthesised faces cannot effectively reproduce the realism of human faces.

Preliminary significance testing was conducted to determine differences in response accuracy between counterbalanced guidance streams (A vs B) *within* each guidance condition (human vs AI). Independent samples t-tests showed no significant differences in response accuracy between the HumanA and HumanB guidance streams (t(152) = 1.47, 95% CI = [-.01, .01], *p* = .14; HumanA mean = .68, HumanB mean = .66) and between the AI_A and AI_B guidance streams (t(139) = -.19, 95% CI = [-.03, .03], *p* = .85; AI_A mean = .66, AI_B mean = .66). Thus, an individual’s ability to correctly identify real and artificial faces as such was not influenced by which of those faces were accompanied by correct or incorrect guidance information. In the statistical analyses, A and B streams were collapsed into one, leaving one human and one AI guidance group.

1. **Response accuracy and consistency.**

Participants made judgements consistent with the guidance information provided more so when that guidance correctly classified faces as real or synthetic. This was true regardless of participant self-reported gender or ethnicity (Table S1).

**Table S1**

*Mean and standard error (SE) consistency scores for stimuli with correct and incorrect guidance, and all stimuli, across participant self-reported gender and ethnicity.*

| **Demographic characteristic** | **Correct (out of 40)** | **Incorrect (out of 40)** | **Correct & incorrect (out of 80)** |
| --- | --- | --- | --- |
|  | **Mean (SE)** | | |
| Gender |  |  |  |
| Male | 29.90 (.32) | 17.70 (.45) | 47.60 (0.58) |
| Woman | 31.00 (.40) | 16.60 (.69) | 47.60 (0.84) |
| Non-binary/ genderqueer/ agender/gender fluid | 27.50 (4.50) | 16.00 (1.00) | 43.50 (5.50) |
| Transman | N/A | N/A | N/A |
| Prefer not to say | N/A | N/A | N/A |
| Ethnicity |  |  |  |
| Asian | 30.00 (1.50) | 20.50 (2.35) | 50.50 (3.52) |
| Black | 30.00 (.67) | 17.80 (.86) | 47.80 (1.20) |
| White | 30.60 (.28) | 17.20 (.46) | 47.80 (0.54) |
| Mixed | 29.10 (1.12) | 13.80 (1.04) | 42.90 (1.69) |
| Other | 27.00 (3.13) | 18.00 (3.13) | 45.00 (4.55) |
|  | **30.30 (.25)** | **17.30 (.38)** | **47.60 (.48)** |

1. **Guidance use level.**

To examine the extent level of guidance use influences response accuracy and consistency differently between guidance groups, boxplots of the interaction between guidance use group for accuracy and consistency data were created (Figure S2). There is no interaction between guidance use type for response accuracy or consistency, nor an effect of guidance stream. These plots show poorer accuracy for the *Always used* group and reduced consistency for the *Did not use* group in both guidance conditions.

A two-way between-subjects ANOVA revealed a significant effect of guidance use level (F(2, 289) = 11.56, *p* < .05), and non-significant effects of guidance stream (F(1, 289) = .81, *p*  = .37) and the interaction between guidance use level and guidance stream (F(2, 289) = .82, *p* = .44) on response accuracy. Pairwise comparisons outputs by Tukey test on guidance use level are identical to those obtained from the one-way ANOVA performed on response accuracy data (Table 3). The same pattern of results was obtained from a second two-way between-subjects ANOVA examining the effect of guidance use level, guidance stream, and the interaction between the two on consistency data. This analysis showed a significant effect of guidance use level (F(2, 289) = 8.33, *p* < .001), but non-significant effects of guidance stream (F(1, 289) = 2.08, *p* = .15) and interaction between guidance use level and guidance stream (F(2, 289) = .30, *p* = .74). As with the first two-way ANOVA, the pattern of pairwise comparisons by Tukey test on guidance use level are identical to those obtained from the one-way ANOVA performed on consistency data (Table 3). These statistical analyses support the conclusions derived from Figure S2.

**Figure S2**

*Boxplots of the interaction between guidance use and guidance stream for response accuracy (top) and consistency data.*

*
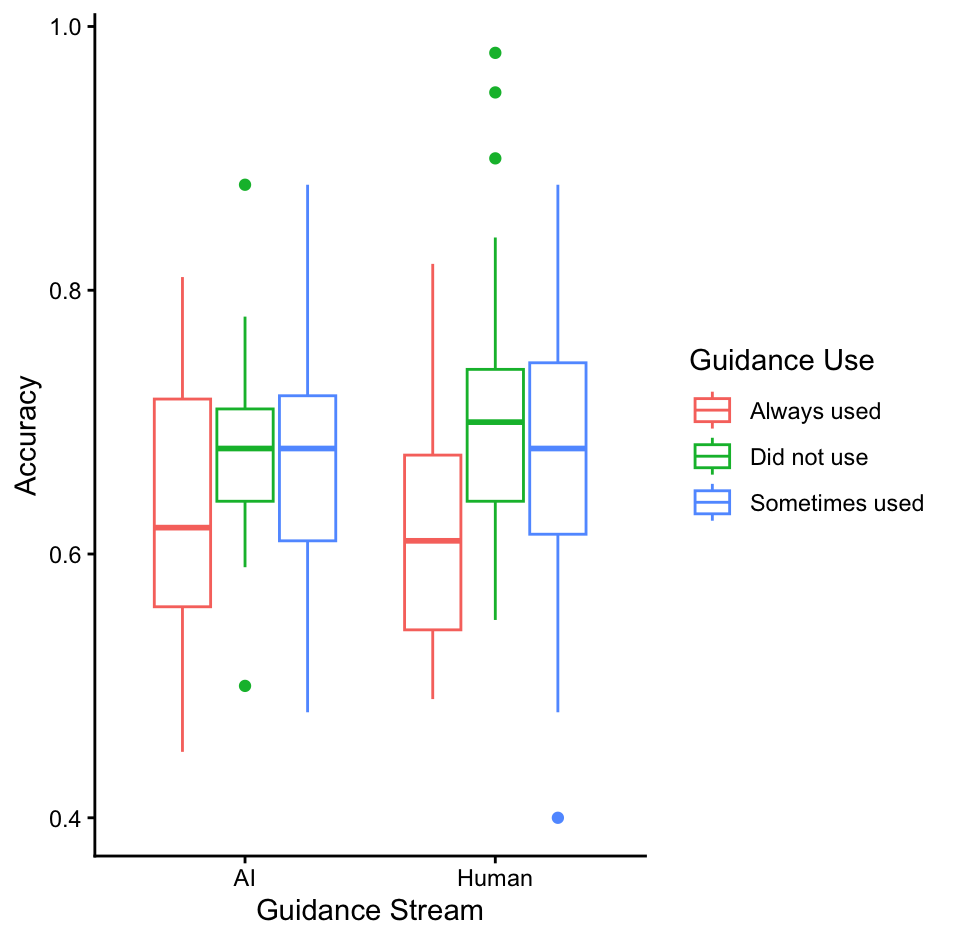
*


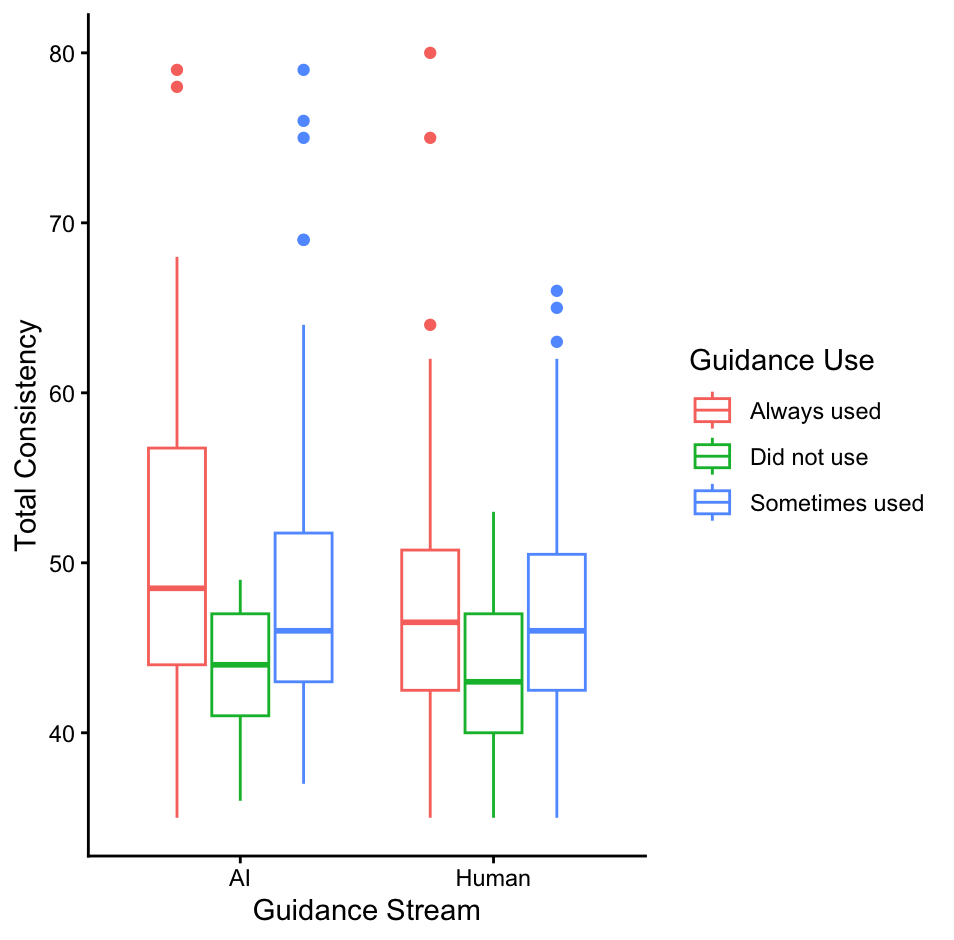


1. **Signal detection analyses.**

**Figure S3**

*Histograms of d’ (top) and c distributions across guidance streams.*

*
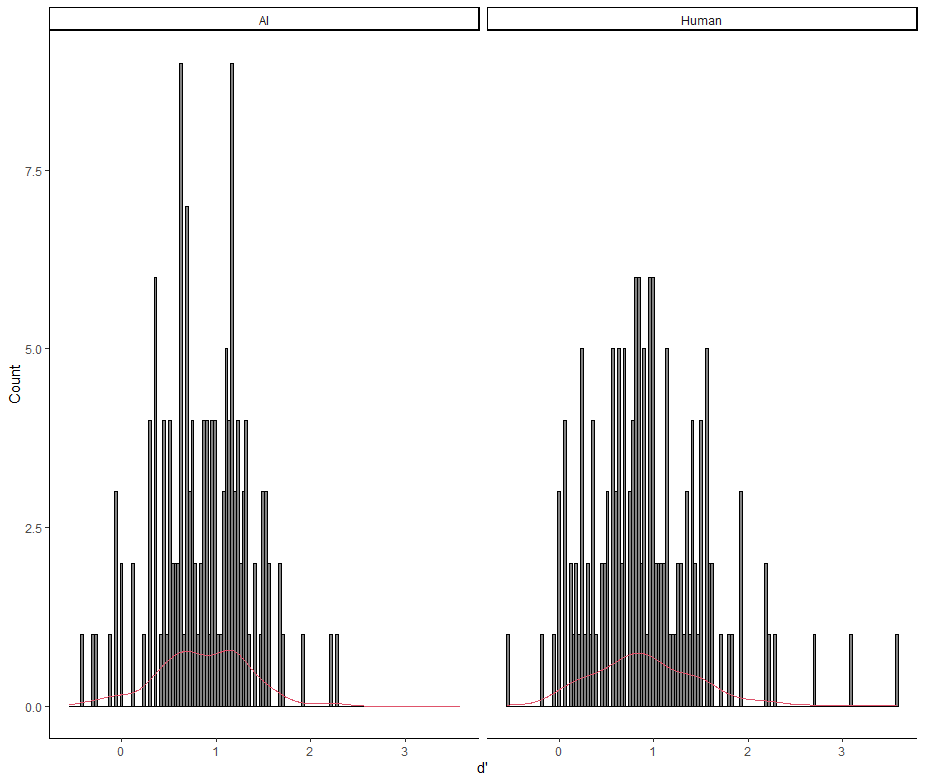

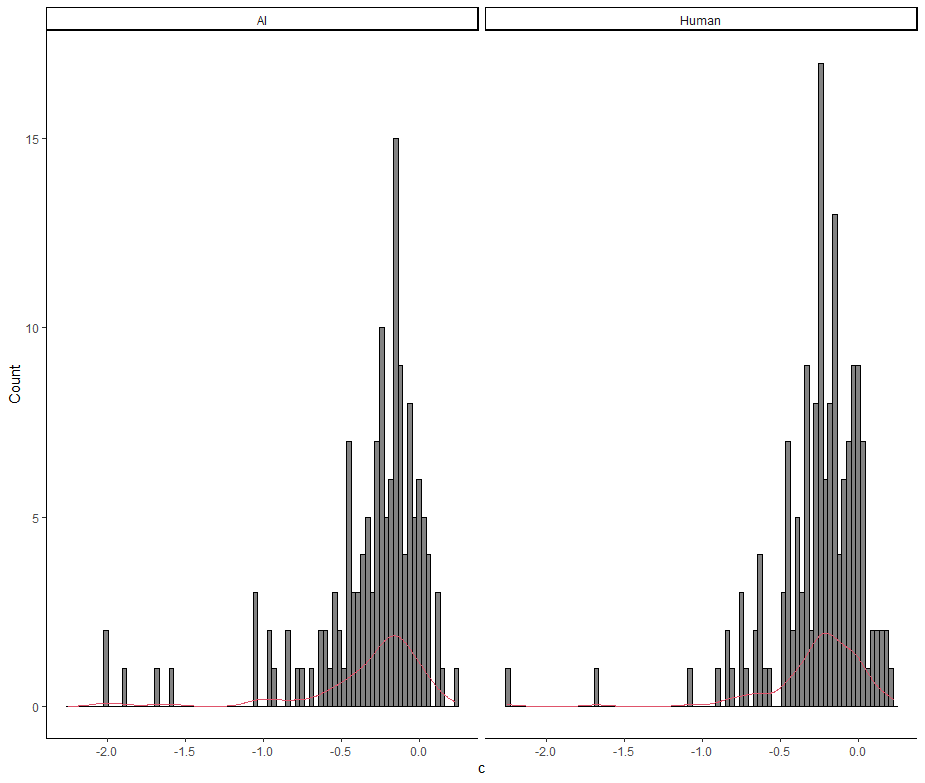
*

To determine if the composite questionnaire used here to examine trust in other humans assessed a latent construct of trust, a CFA using maximum likelihood estimation was conducted. The model specified one latent variable (trust) underlying all observed indicators (excluding item 11, an attention check). Model fit was determined by examining: Chi-square (X^2^), a measure of overall model fit; Root Mean Square Error of Approximation (RMSEA) and Standardised Root Mean Square Residual (SRMR), measures of how far a model is from perfect fit; Tucker-Lewis Index (TLI) and Comparative Fit Index (CFI), which compare model fit to the worst possible model. The model demonstrated poor fit to the data, indicated by a significant X^2^ test (X^2^(119) = 967.20, *p* < .001). TLI and CFI scores of .69 and .73 fall below the commonly accepted threshold of .90 for adequate fit, while RMSEA and SRMR values of .16 and .09 exceed the typical cutoff scores of .08. Together, these results indicate that a one-factor structure does not adequately represent the data.

To rectify this, low-loading items (Table S2) were removed from a second CFA specifying a single latent construct of trust. The resulting 15-item model remained a poor fit for the data: X^2^(90) = 881.35, *p* < .001; CFI = .741; TLI = .698; RMSEA = .173, 90% CI [.162, .183]; and SRMR = .096. Despite the removal of two low-loading items, the one-factor structure remained an inadequate representation of the data.

**Table S2**

*Factor loadings and standard error (SE) for CFA of 17-item composite human trust scale.*

| **Questionnaire item** | **Factor loading (SE)** |
| --- | --- |
| human_trust_scale_1 | .89 (.05) |
| human_trust_scale_2 | .88 (.05) |
| human_trust_scale_3 | .79 (.05) |
| human_trust_scale_4 | .63 (.05) |
| human_trust_scale_5 | .88 (.06) |
| human_trust_scale_6 | .56 (.05) |
| human_trust_scale_7 | .54 (.05) |
| **human_trust_scale_8** | **.33 (.05)** |
| **human_trust_scale_9** | **.19 (.05)** |
| human_trust_scale_10 | .71 (.06) |
| human_trust_scale_12 | .54 (.05) |
| human_trust_scale_13 | .80 (.06) |
| human_trust_scale_14 | .57 (.05) |
| human_trust_scale_15 | .71 (.06) |
| human_trust_scale_16 | .66 (.06) |
| human_trust_scale_17 | .74 (.06 |
| human_trust_scale_18 | .86 (.06) |

*Note.* Items in bold load weakly onto the latent construct and, therefore, were identified as problematic (Brown, 2015).

A linear regression with *d*$'$ as dependent variable and GAAIS positive and negative subscale scores, human trust scale score, and self-reported level of guidance use as independent variables was performed. The previously identified effect of GAAIS negative subscale on task performance was preserved, such that a one unit increase in negative attitudes towards AI yielded a .13 (*p* = .008) unit decrease in *d*$'$*.* No effect of GAAIS positive subscale or human trust scale scores were observed. A significant effect of self-reported guidance use was observed, such that moving from *Always used* to *Did not use* yielded a .38 (*p* < .001) unit increase in *d*$'$ value and from *Always used* to *Sometimes used* a .26 (*p* < .001) unit increase in *d*$'$ value. Lower self-reported guidance use predicts an improved ability to discriminate between real and synthetic faces. ANOVA revealed a significant improvement in model fit (*p* < .001) with the inclusion of guidance use level.

The same linear regression but with *c* as the dependent variable revealed a significant effect of self-reported guidance use level, such that moving from *Always used* to *Did not use* yields a .26 (*p* < .001) unit increase in *c* value, indicating that less reliance on guidance predicts a reduced likelihood of classifying faces as real. The previously observed non-significant effects of GAAIS subscales on *c* score persisted but a new effect of human trust scale on *c* score was observed, such that a one unit increase in trust in humans yields a .07 (*p* = .03) decrease in *c* value. Greater trust in humans predicts a greater tendency to classify faces as real when the effect of guidance use is controlled. ANOVA showed a significant improvement in model fit (*p* < .001) with the inclusion of guidance use level.

A linear regression with *d*$'$ as dependent variable and GAAIS positive and negative subscale scores, human trust scale scores, self-reported level of guidance use, and guidance stream as independent variables was performed. No significant effect of guidance stream on *d*$'$ was identified, while the pattern of results for GAAIS subscales, human trust scale, and self-reported guidance use level remained identical to that observed in the previous model. The significant effects of GAAIS negative subscale (*b* = -.14, SE = .05, *p* = .007) and guidance use level – participants who did not use or sometimes used the guidance showed significantly larger *d*$'$ scores than those who always used it (*b* = .37, SE = .10, *p* < .001 and *b* = .25, SE = .08, *p* = .001 respectively) – were preserved. Thus, more positive attitudes towards AI and less self-reported guidance use predicted an improved ability to discriminate between real and synthetic faces. Furthermore, ANOVA revealed a non-significant (*p* = .31) improvement in model fit with the inclusion of guidance stream. The same regression model was fit with *c* score as the dependent variable. No significant effect of guidance stream on *c* score was observed. The pattern of results for the remaining independent variables were identical to those identified in the previous modelling iteration. The significant effects of human trust scale (*b* = -.05, SE = .02, *p* = .009) and guidance use level – participants who did not use the guidance showed significantly larger *c* values than those who always used it (*b* = .26, SE = .07, *p* < .001) – were preserved. Thus, a greater propensity to trust other humans predicted a greater tendency to classify faces as real, while less self-reported reliance on the available guidance information predicted a reduced likelihood of classifying faces as real. ANOVA revealed a non-significant (*p* = .16) improvement in model fit with the inclusion of guidance stream.

Two final regression models accounting for the influence of participant sociodemographic characteristics on *d*$'$ and *c* scores were created. These additional parameters were introduced as independent variables into the most complex model identified by ANOVA as yielding a significant improvement in model fit from its predecessor. Thus, linear regressions with *d*$'$ and *c* as dependent variable and GAAIS positive and negative subscale scores, human trust scale scores, guidance use level, age, gender (Man, Woman, Non-binary/genderqueer/agender/gender fluid, Transman, prefer not to say), and ethnicity (Asian, Black, Mixed, White, Other) as independent variables were constructed.

Non-significant effects of human trust scale and GAAIS positive subscale scores on d’ were observed. A significant effect of GAAIS negative subscale score on *d*$'$ was observed (*b* = -.11, SE = .05, *p* = .03). Thus, more positive attitudes towards AI predicted a reduced ability to discriminate between real and synthetic faces. A significant effect of guidance use was observed, such that participants who did not use or sometimes used the guidance showed significantly greater *d*$'$ scores than those who always used it (*b* = .30, SE = .10, *p* = .004 and *b* = .19, SE = .08, *p* = .02 respectively). Additionally, significant effects of participant age (*b* = -.01, SE = .00, *p* < .001) and gender (*b* = .17, SE = .07, *p* = .01) on *d*$'$ scores were observed. Thus, younger participants and women showed an increased ability to discriminate between real and artificial faces. Participant ethnicity did not significantly predict *d*$'$ score. A significant effect of human trust scale (*b* = -.04, SE = .02, *p* = .04) on *c* values was observed, such that a greater propensity to trust other humans predicted an increased likelihood to classify faces as ‘real’. A significant effect of guidance use was observed, such that participants who did not use the guidance showed significantly larger *c* values than those who always used it (*b* = .24, SE = .07, *p* < .001). A significant effect of participant age (*b* = -.004, SE = .002, *p* = .04) on *c* values was observed. Thus, older participants showed an increased likelihood of classifying faces as ‘real’.
